# The Relative Burden of Occupational Injuries and Illnesses in Firefighters: An Analysis of Washington Workers’ Compensation Claims, 2006–2020

**DOI:** 10.3390/ijerph20227077

**Published:** 2023-11-17

**Authors:** Naomi Anderson, Jennifer Marcum, David Bonauto, Miriam Siegel, Claire LaSee

**Affiliations:** 1 SHARP Program, Washington State Department of Labor and Industries, Olympia, WA 98504-4000, USA; jennifer.marcum@lni.wa.gov (J.M.); david.bonauto@lni.wa.gov (D.B.); claire.lasee@lni.wa.gov (C.L.); 2 Division of Field Studies and Engineering, National Institute for Occupational Safety and Health (NIOSH), Centers for Disease Control and Prevention (CDC), Cincinnati, OH 45226, USA; wrm9@cdc.gov

**Keywords:** firefighters, law enforcement officers, workers’ compensation claims, first responder, occupational injuries

## Abstract

Firefighters face many hazards on the job and have a high rate of work-related injuries and illnesses (WRII). We analyzed Washington workers’ compensation claims from 2006–2020 to characterize WRII in firefighters compared to law enforcement officers and “all other” workers. There were 9187 compensable claims for firefighters, 7801 for law enforcement officers, and 586,939 for “all other” workers. Nearly 40% of claims for firefighters were work-related musculoskeletal disorders (WMSDs). The claim rate per 10,000 full-time equivalent (FTE) firefighters was 716.4, which is significantly higher than that of law enforcement officers (510.0) and “all other” workers (163.2). The rate per 10,000 FTE of WMSD claims was also higher in firefighters (277.0) than in law enforcement officers (76.2) and “all other” workers (57.6). Additional WRII among firefighters commonly included being struck or caught in objects, slipping or tripping, and exposure to caustic or noxious substances; and amongst law enforcement, transportation accidents and violence. Medical costs and time-loss days per claim were lower for firefighters and law enforcement than for “all other” workers. Common tasks associated with WMSDs in firefighters included lifting and transporting patients, using specific tools and equipment, and physical training. WMSDs stand out as an area for prevention and intervention activities.

## 1. Introduction

Firefighters routinely engage in duties that expose them to a range of safety and health risks [1,2,3]. Firefighting and emergency medical response are especially physically demanding work activities, requiring frequent lifting, pushing, and pulling of heavy loads that place firefighters at an increased risk for work-related musculoskeletal disorders [1,4,5]. Such work is often performed wearing heavy gear and equipment and in work environments that further aggravate the risk for injury. These environments can range from the cramped spaces of an aid car or nursing home bathroom to emergency rescues of victims trapped in crashed vehicles or burning buildings. Working in these conditions can manifest in awkward postures and unusual physical loading of the musculoskeletal system. Evaluation of workers’ compensation claims supports the supposition that firefighters are at higher risk of work-related musculoskeletal disorders. In Washington State (WA), firefighters have been identified as one of the top three occupational groups in need of work-related musculoskeletal disorder (WMSD) prevention when comparing the count and rate of wage replacement workers’ compensation claims [5].

Despite these data, in WA, interest in preventing musculoskeletal disorders emerged indirectly from proactive efforts to control firefighter exposure to carcinogens. Like many states in the US, Washington State considers certain medical conditions to be legally presumed as occupational diseases for the purposes of workers’ compensation, including specific cancers, respiratory disease, certain heart problems, specific infectious diseases, and posttraumatic stress disorder (PTSD) [6]. Periodic updating of WA’s firefighter presumption law transitioned legislative interest from not only providing compensation for firefighters with specified disease outcomes, but to provide workers’ compensation incentives and grants to fire departments for adoption of best practices [7] to prevent carcinogen exposure. Providing fire departments with incentives to identify and adopt best practices for carcinogen exposure also provides the opportunity for fire departments to engage more holistically in identifying and adopting best practices for other work tasks known to create risk for firefighter injury and illness. Furthermore, a transformational approach to safety would apply a continuous process of proactively identifying, evaluating and controlling safety and health hazards, specifically, a risk management system [8]. Published research using risk management in the fire service has led to injury reduction and workers compensation savings [1]. Thus, in 2019, WA passed legislation [9] to allow workers’ compensation incentives and an equipment grant program for fire departments to adopt a risk management model to identify and adopt best practices to prevent carcinogen exposure and to more broadly prevent other common causes of compensable workers’ compensation claims.

In support of these legislative efforts to promote more comprehensive risk management, we examined firefighters in the Washington State workers’ compensation system to understand overall work-related injury and illness burden, including both WMSDs and other conditions. We aim to describe the most common causes of injury and illness among firefighters in WA. Furthermore, because WMSDs are a known problem [5] for firefighters in Washington State, we sought to further characterize these claims in more detail, including by demographics of the injured worker and injury circumstances. This information can be used to help prioritize best practices within the risk management framework as described above. Comparison data are provided to highlight how risks to firefighters compare to other workers in general. Law enforcement officers are also provided as a comparison group to better control for confounding, like physical fitness and demographics, and to isolate the workplace hazards unique to firefighting.

## 2. Materials and Methods

### 2.1. Workers’ Compensation System

Washington (WA) State is one of four US states with an exclusive workers’ compensation system [10] in which the insurance coverage is provided primarily or exclusively by the state. In WA, employers must obtain workers’ compensation coverage through the WA Department of Labor and Industries (L&I) [11] state fund, unless they meet specific financial and safety requirements in order to self-insure [12]. Typically, only the larger employers meet these criteria to self-insure. The majority of workers in WA are covered by the state fund, and the rest (approximately 25%) [13] are covered by their employers through self-insurance. WA L&I administers the state fund and regulates the management and administration of self-insured employers’ claims.

Data from both state fund and self-insured claims are entered and stored in the WA L&I Industrial Insurance Data Warehouse, along with data on employers and employee hours worked. Self-insured claims are regulated by and reported to WA L&I, but are administered by a third-party administrator. Claims suggested for rejection by a self-insured employer’s third-party administrator are reviewed by WA L&I for a final determination. Self-insured employers are not required to report claim costs or time loss days to WA L&I, therefore claim costs and time loss information can only be analyzed among state fund claims. Unless otherwise noted, all results include both state fund and self-insured data.

Workers who are injured or made ill at work initiate a workers’ compensation claim with their medical provider when seeking care by completing a Report of Industrial Injury or Occupational Disease form to WA L&I or their self-insured employer. Information on this form is available for both state fund and self-insured claims, and includes injured worker demographic and contact information; information about their employer; their job title; and the circumstances of the injury or illness. The Report of Industrial Injury or Occupational Disease form also contains a section for the medical provider to provide information on the initial injury or illness diagnoses, and an assessment of a causal relationship to work.

Claims may be accepted or rejected. If accepted, claims are further categorized as either medical-aid only or compensable. Medical-aid only claims are typically less severe and do not result in more than 3 days away from work. For these claims, only medical costs are covered by workers’ compensation. “Compensable” claims have additional costs beyond medical care and include those that progress to wage-replacement (time loss/indemnity payment), permanent disability, kept on salary, loss of earning power, and/or death. Compensable claims generally have greater injury severity than medical-aid only claims. To qualify as compensable, the worker must be unable to work for more than a three-calendar day waiting period. Only compensable claims were analyzed for this study.

### 2.2. Case Identification and Extraction

Claim data were extracted for this cross-sectional study on 16 February 2023. Claims were restricted to those with an injury date between 1 January 2006 and 31 December 2020.

Three distinct occupational groups (firefighters, law enforcement officers, and “all other” workers) were defined for this analysis according to WA risk classification codes. Risk classes are coded according to a WA-specific classification system that groups exposure by risk for insurance purposes [14]. In WA, employers pay their workers’ compensation premiums based upon hours worked by risk classification. Firefighters were identified by the “county and city firefighters—salaried” risk class, and law enforcement officers were workers in the “county and city law enforcement officers” risk class. For additional comparison, data were extracted for “all other” workers and includes all workers except firefighters and law enforcement officers as previously defined. Volunteer firefighters are not covered by the WA L&I workers’ compensation system and are therefore not included in this analysis. In WA, volunteer firefighters have a separate disability and death insurance benefit system administered by the Volunteer Firefighters’ and Reserve Officers Relief and Pension Act (RCW 41.24). According to the U.S. Fire Administration Fire Department Registry, 47% of active firefighters in WA departments are volunteer firefighters [15].

### 2.3. Injury and Illness Characteristics

Following workers’ compensation claim submission, trained coders assign Occupational Injury and Illness Classification System (OIICS) v1.01 [16] codes to characterize the injury or illness event type (e.g., “fall to lower level”), nature (e.g., “sprains, strains, tears”), source (e.g., “hand tools-nonpowered), and body part affected. For this analysis, claims were grouped into mutually exclusive injury and illness event types based on the assigned OIICS codes. Most injury or illness groupings are presented using the OIICS v1.01 nomenclature, and then aggregated to highlight common causes for firefighters. WMSDs were an exception and were identified using a combination of OIICS nature and event codes developed for a previously established WA definition. The details on the WMSD definition are described elsewhere [5]. In general, WMSDs are injuries or conditions that disrupt the musculoskeletal system caused by certain work activities such as forceful exertion, repetitive motion, awkward postures, prolonged sitting or standing, or vibration.

Additionally, data on COVID-19 and PTSD claims are presented. These conditions are not readily identified within the OIICS v1.01. Claims for occupational exposure to the COVID-19 virus were identified and validated via a method established and previously described by the respiratory disease surveillance project at the Washington State Dept. of Labor and Industries using OIICS codes, ICD-9-CM diagnosis codes, and keywords [17]. COVID-19 claims are only described for 2020.

Characterizations of cancer and other illnesses that are legally-presumed to be occupational diseases in WA firefighters have been previously described in detail [6]. Therefore, while claims for these conditions are included in the data (counted in the total frequencies; included in the “all other” category in the presentation of results by injury type), most of these conditions are not examined separately in this analysis. Post-traumatic stress disorder (PTSD) is the exception and has been given increased consideration in regards to potential presumptive coverage in WA in recent years. A presumptive coverage flag was added for PTSD claims in the WA workers’ compensation system in 2018 and was used to identify PTSD claims there. These data should be interpreted with caution, however, as this flag may not have been used consistently since it was first added to the workers’ compensation system. As another potential measure for PTSD, we also present claims for Post-Traumatic Anxiety (PTA) as identified by OIICS v1.01 codes for Nature of Injury or Illness 5211 and 5212: “post-traumatic anxiety-acute” and “post-traumatic anxiety-chronic”, respectively (OIICS v1.01 coding does not include a code specifically for PTSD). Under the existing presumptive coverage statute, firefighters and law enforcement officers are eligible to file for workers’ compensation when PTSD/PTA is the primary diagnosis. However, other occupations are excluded from such coverage, so we do not provide these data for the “all other” occupation group.

WMSDs are of particular interest for the firefighter population, so we have provided additional information for these claims. Specifically, we described the distribution of the OIICS source of injury or illness for WMSD claims. We also abstracted free-text information from the ‘injury description’ field on the Report of Injury or Occupational Disease form to provide some common tasks or situations associated with the compensable WMSD claims for specific sources of injury among firefighters.

### 2.4. Statistical Analyses

Descriptive analyses were conducted to characterize the burden of work-related injury or illness among firefighters, law enforcement officers, and “all other” workers. We characterized claims by the following characteristics: insurer type (state fund vs. self-insured); claimant demographics (gender, age, marital status), injury event type; select injury natures; injury time of day and season; claim costs (state fund claims only); and time loss (for claims with a reported loss time of >0 days; state fund claims only) for each occupational category. Average number of compensable claims per claimant was also calculated to determine if firefighters are more or less likely to have multiple compensable claims compared to the other groups. Workers may have other claims, e.g., medical only or rejected claims, which are not included in this calculation.

Claim costs for closed claims are those paid to date on the claim. For claims open at the time of data extraction (approximately 3% of each occupational group), claim costs are those paid to-date on the claim and the actuarial case reserve estimate for future claim costs. State fund claims were analyzed for medical costs alone, and for total claim costs (including medical costs, indemnity payments, and all other costs incurred by the claim). Costs were not adjusted for inflation.

We calculated claim rates per 10,000 full-time equivalent (FTE) workers using the frequency of claims divided by the sum of FTEs calculated for each occupational group by injury type and/or year. Because workers’ compensation premiums are determined by hours worked in each risk class, we were able to calculate specific denominators for each occupational group evaluated here (reported hours/2000 = 1 FTE).

Claim rates are presented with their respective 95% confidence intervals to provide information on stability of the estimate. Claim rate trends over time were analyzed using negative binomial regression to account for overdispersion in count data, controlling for reported hours (FTE). Changes in trend were considered statistically significant for *p*-values less than 0.05; where injury rates are presented by injury type, we defined statistically significant differences as existing where the 95% confidence intervals between the rate estimates did not overlap.

All analyses were conducted using SAS 9.4 (SAS Institute, Inc., Cary, NC, USA). The study was conducted by the Washington State Department of Labor and Industries, a public health authority with statutory obligations to compile statistics and data for the control of occupational injuries and illness in Washington State. This study was approved by the Washington State Institutional Review Board (WSIRB).

## 3. Results

Table 1 presents the annual averages for number of full-time equivalents, number of workers’ compensation compensable claims, and compensable claim rates per 10,000 FTE, by insurer type (state fund or self-insured) for firefighters, law enforcement, and “all other” workers during 2006 through 2020. The state fund covers about three quarters of all WA workers, including the majority (61.4%) of firefighters, Table 1. During the study period, the proportion of firefighters covered by the state fund was increasing (from 56% in 2006 to 65% in 2020). The majority (63.4%) of law enforcement officers are employed by self-insured entities (Table 1) and this proportion remained consistent over time (from 63% in 2006 to 64% in 2020). The state fund and self-insured combined compensable claim rate was higher among firefighters compared to law enforcement officers and “all other” workers (716.4 vs. 510.0 vs. 163.2 per 10,000 FTE, respectively), Table 1. This was also true when comparing across occupations for state fund only claim rates, and self-insured claim rates. Self-insured compensable claim rates were consistently higher than state fund rates in all three occupational groups (approximately 2 times higher among firefighters and 1.5 times higher for law enforcement officers and “all other” workers), Table 1.

Demographic characteristics of claimants are presented by occupational group in Table 2. Firefighters had a slightly higher number of compensable claims per claimant compared to law enforcement officers and “all other” workers (1.8 vs. 1.5 and 1.3, respectively). Firefighters and law enforcement officer claimants were distinct from “all other” worker claimants for having higher proportions of male workers (Table 2). “All other” workers had higher proportions of workers in both the younger and oldest age categories compared to firefighters and law enforcement officers (Table 2), which may reflect the minimum physical and legal requirements for employment as a firefighter or law enforcement officer and earlier separation from firefighting and law enforcement work due to retirement benefit eligibility. Firefighter and law enforcement officer claimants were more likely to be married than “all other” claimants.

Most injuries that led to compensable claims happened between 6:00 a.m. and 5:59 p.m. for all occupational groups, Table 3. However, law enforcement officers had more compensable claims for injuries that occurred between 6:00 p.m. and 11:59 p.m. than firefighters and “all other” workers, Table 3. There was little variation in season among all groups, Table 3.

Table 4 presents the distribution and rates of compensable claims for analyzed injury types. The most common injury type for firefighters was WMSDs, accounting for 39% of compensable claims (Table 4), and which occurred at a significantly higher rate than in law enforcement and “all other” workers. While firefighters had higher rates than law enforcement and “all other” workers for the majority of injury types, law enforcement officers had the highest rates for “fall to same level”, and higher rates of “transportation accidents” and “assaults and violent acts” injuries than both firefighters and “all other” workers, Table 4. Both firefighters and law enforcement officers had higher claim rates and proportions of work-related illnesses and injuries classified as “bodily reaction” than “all other” workers. Additional analyses revealed that, for “bodily reaction” claims in these data, the body part most commonly affected was the knee. Approximately 32% of these claims in both FF and law enforcement officers involved the knee with the common exposure being from walking, running, and/or stepping in heavy gear, on uneven or hazardous surfaces, and while engaged in training operations or with law enforcement officers in pursuit .

Other conditions of interest that reflect the unique occupational hazards encountered by firefighters are presented in Table 5, including compensable claims for COVID-19 exposure, PTSD and PTA. Firefighters had a substantially higher rate of COVID-19 in 2020 (321.7 per 10,000 FTE), Table 5. Firefighters and law enforcement had similar rates of post-traumatic stress/anxiety, Table 5.

Claim costs and time-loss days are compared across occupational groups for state fund claims in Table 6. As compared to both law enforcement officers and “all other” workers, firefighters had lower overall costs and lower medical costs, for all claims and for WMSDs. For days of time-loss, firefighters had a very similar distribution to law enforcement officers, but less time-loss compared to “all other” workers. The data for claim costs and time-loss days were highly skewed, with a small number of expensive claims with long time-loss affecting the distribution among all occupational groups, Table 6.

Details on the source of injury or illness for compensable WMSD claims in firefighters, along with common tasks taken verbatim from the injury descriptions are presented in Table 7. Interacting with a “healthcare patient or resident of a healthcare facility” was the leading Source of WMSDs in firefighters, Table 7. Other common sources were equipment, such as “hoses”, “ladders”, and “truck”, Table 7. Of claims with a source of “no description at this level”, 60% were self-insured claims. 

**Figure 1:**
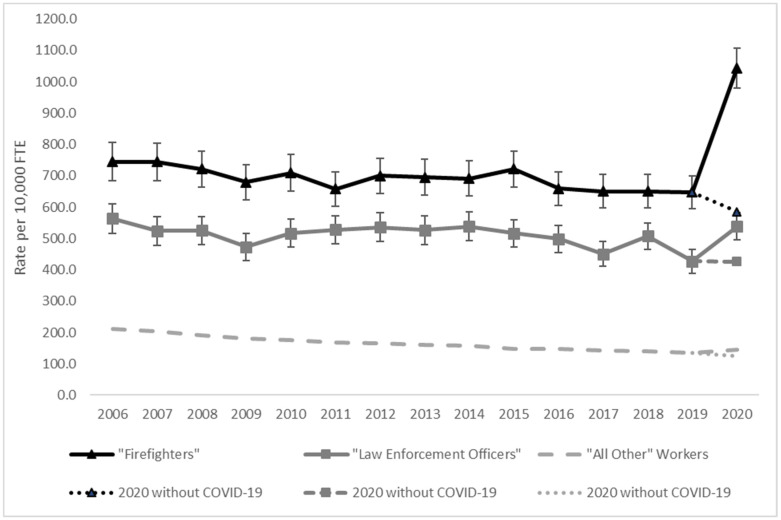
Compensable ^1^ claim rate trend by occupational group, Washington State, 2006–2020. ^1^ Compensable claims are those that result in wage replacement/time loss, kept on salary, disability, or fatality.

**Figure 2:**
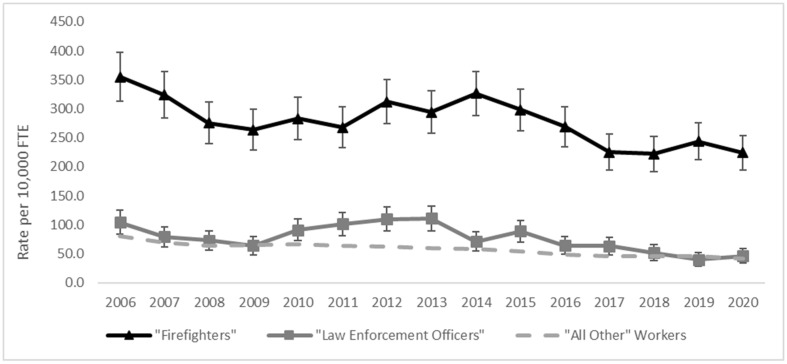
Compensable ^1^ Work-Related Musculoskeletal Disorder (WMSD) ^2^ claim rate trend by occupational group, Washington State, 2006–2020. ^1^ Compensable claims are those that result in wage replacement/time loss, kept on salary, disability, or fatality. ^2^ Work-related musculoskeletal disorders (WMSDs) definition-based OIICS nature and event codes v1.01. See Ref [5].

Figure 1 and Figure 2 show the compensable claim rates of all work-related injuries or illnesses (Figure 1) and WMSDs only (Figure 2) for firefighters, law enforcement officers, and all other workers by year. Yearly compensable claim rates for firefighters were significantly higher than rates for law enforcement officers and “all other” workers throughout the study period. Firefighters had a noticeable increase in compensable claims in the year 2020—almost twice the yearly average of 2006–2019—due to COVID-19 claims.

The trend of compensable claim rates of all work-related injuries or illnesses was significantly different for firefighters and law enforcement officers compared to “all other” workers during 2006 through 2020, Figure 1. Firefighters and law enforcement officers had no change in claim rate by year during the time period. In contrast, “all other” workers had an estimated 3.0 percent decrease per year in claim rate (*p* < 0.0001), Figure 1. When restricting to years 2006–2019 (excluding the increase in 2020), firefighters had a 0.09 percent decrease in claim rates per year (*p* < 0.0008); and law enforcement officers had a 1.0 percent decrease per year (*p* < 0.004). For WMSD claims, all occupational groups had annual decreases in the compensable claim rates during the study time-period. Specifically, the rate for firefighters decreased an estimated 2.0 percent per year (*p* < 0.0001), and law enforcement officers and “all other” workers had an estimated 4.0 percent decrease per year (*p* < 0.001, *p* < 0.0001, respectively), though the annual rates for firefighters were still higher than those of the other groups, Figure 2.

## 4. Discussion

Firefighting and law enforcement are both first-response occupations in which workers regularly face hazardous situations. In this analysis, injured firefighters were similar to injured law enforcement officers in terms of demographic characteristics, claim costs, and time loss duration. The compensable claim rates were substantially higher among firefighters and law enforcement officers compared to “all other” workers. Compensable claims for firefighters and law enforcement officers were often caused by different events. WMSDs were the leading cause of compensable claims among firefighters and “all other” workers. Firefighters were more likely to have injuries or illnesses from being struck or caught in objects, slipping or tripping, and exposure to caustic or noxious substances, while law enforcement officers were more likely to be in transportation accidents or injured from violence. This highlights the unique nature of tasks and exposures in firefighting as a first-response occupation.

Using law enforcement officers as a comparison group for firefighters is a reasonable approach in firefighter safety and health research, given that both are predominantly male workers, and each occupation maintains an expectation of physical fitness on entry into employment. Therefore, by comparing the two groups, we controlled for some residual confounding that we were unable to control for when comparing to a general working population. Nonetheless, our analyses allowed us to also identify injuries and illnesses for which law enforcement officers may also be vulnerable, including falls on same level, motor vehicle incidents, violence, and PTSD.

The gender distribution in our workers’ compensation data is comparable to the gender distribution in employment estimates for firefighters and law enforcement officers [18,19,20,21,22]. Female firefighters and law enforcement officers accounted for 8.2% and 13.2% of compensable claims, respectively. Active membership data from the WA Law Enforcement Officers’ and Fire Fighters’ (LEOFF) retirement system reports 6% of employed firefighters and 10% of employed law enforcement officers as female in 2019 [21]. Therefore, risk of a compensable claim does not appear to vary substantially by gender among firefighters and law enforcement officers as it does for “all other” workers. In comparison, data from the Employed Labor Force (ELF) query system for the years 2015–2019 yielded an estimate of 53.8% male for WA workers when excluding police and firefighting occupations [23].

Firefighters also had a higher average number of compensable claims per claimant, which may indicate that firefighters are being injured more often, or suggest that they have better knowledge of and access to workers’ compensation systems. This may be especially true when comparing firefighters to “all other” workers, which includes workers in many occupations that may be facing barriers to filing claims [24]; union membership [25,26], race/ethnicity [25,27], wages [25,28], immigration status [26,29], and other personal, social and economic factors [24,25,26,27] may affect claim filing.

The number of workers’ compensation claims for firefighters were declining over time in a recent study of firefighters in Ohio [19]. Our study found a decline in claim rates per 10,000 FTE over the study period, if excluding COVID-19 claims in 2020; the rate for WMSDs was also declining for all groups. Work in California found that injury rates, particularly for musculoskeletal disorders, were very high in firefighters [30] compared to other workers, and with similar patterns in regards to police officers. Firefighters also had “the highest share of injuries that are musculoskeletal in nature” (47%—firefighters, 38–police officers) [30].

Firefighters and law enforcement officers had shorter time loss durations than “all other” workers for compensable claims. This observation in the context of research regarding predictors of chronic disability is intriguing. Both firefighters and law enforcement officers enter into emergency response situations where there is threat to their personal safety and health. In a prospective study of Washington workers’ compensation claimants with low back pain in the general working population, after adjusting for demographics, pain, disability, and other psychosocial variables, significant predictors of chronic disability at six months following injury were “fear avoidance” or beliefs that work may increase or cause pain, and having low recovery expectations after injury [31]. Another psychosocial consideration regarding likelihood of chronic disability [32] involves pain catastrophizing, which is “characterized by the tendency to magnify the threat value of pain stimulus and to feel helpless in the context of pain, and by a relative inability to inhibit pain related thoughts in anticipation of, during, or following a painful encounter” [33]. Furthermore, both firefighters and law enforcement officers each exist within a work environment where organizational and social support systems may motivate the worker to return to work [34]. An analysis of firefighters in Ohio found that there was some injury seasonality in claims (a higher proportion of slip/trip/fall injuries in the winter months) [19], though our data did not demonstrate much variability across seasons.

A study of firefighters and emergency responders in Ohio found that “overexertion” was the most common cause of injury for emergency personnel with injury claims involving a shoulder, low back, or knee [35]. Among the Ohio sample of claims analyzed, costs varied by body-part injured. Furthermore, firefighters in the Ohio study, as compared to police officers and police leaders, had lower indemnity costs, medical costs, and total lost days per claim [35], which is similar to our results.

In a study of two large U.S. metropolitan fire departments, the authors found ”overexertion and bodily” to be the most commonly reported cause of injury (54.1%) and therefore, suggest “a combined initiative that promotes enhanced fitness and ergonomics based on a careful analysis of the physical demands of firefighting in tandem with the use of tools and equipment” [4]. In our analysis, WMSDs were the leading injury type among firefighters, with a rate that was significantly higher than that of law enforcement officers and “all other” workers—a pattern that was consistent across study years. In our analyses, we were able to highlight some of the uniquely difficult and hazardous conditions navigated by firefighters during their daily work. For example, firefighters have many patient-handling exposures, which are specific to the occupation. Common situations described in the injury descriptions of the WMSD claims in this analysis were lifting and transferring patients, using occupation-specific tools and equipment, and physical training. Firefighters are unable to control the location and layout of their emergency calls; for example, if they are lifting patients in a nursing home, there may be safe patient handling equipment available, but if they are extricating a patient from a hazardous location, equipment, time, and positional options may be constrained by the life-threatening conditions at hand.

Firefighters had an unusually high number and rate of claims in 2020, reflecting the impact of COVID-19. As supported in our analysis, firefighters have significant exposure to the public, including residents in healthcare and nursing facilities, which had particularly acute COVID-19 infection rates during 2020. With the onset of the COVID-19 pandemic, in March 2020, Washington’s governor authorized policies to allow first responders and healthcare workers to receive workers’ compensation wage replacement when quarantined after a COVID-19 exposure [36]. Additionally, if the worker became ill from COVID-19, their illness was categorized as an occupational disease for purposes of workers’ compensation, and the first responder was entitled to medical and indemnity benefits [36]. This presumptive coverage for COVID-19 claims was not extended to all occupations, and therefore, when comparing rates of COVID-19, firefighters and law enforcement would be expected to have higher rates of compensable claims for COVID-19 than other, non-covered occupations. While both occupations were covered, firefighters had a compensable claim rate that was almost three times higher than that of law enforcement officers. Injury event descriptions from the firefighter claims for COVID-19 mentioned both more routine exposures, such as coworkers and specific occupational exposures, such as responding to nursing homes calls and performing cardiopulmonary resuscitation or other life-saving medical treatments on known COVID-19 positive patients. 

The rates of post-traumatic stress disorder (PTSD) and post-traumatic anxiety for firefighters and law enforcement officers in these data likely result from new benefits in Washington workers’ compensation law. In 2019, with amendments to Washington’s firefighter occupational disease presumption law (RCW 51.32.185 [37]), the diagnosis of PTSD in firefighters and law enforcement officers was presumed to be an occupational disease and eligible for workers compensation benefits. While all other workers are eligible for treatment and care for mental health disorders as a sequelae of an occupational injury or illness, firefighters and law enforcement officers can initiate a claim where PTSD is the sole reason for initiating a workers’ compensation claim.

### 4.1. Limitations

There are several limitations to using workers’ compensation data to examine the burden or work-related injury and illness in specific occupations. Workers’ compensation data does not provide complete estimates of work-related injury or illness due to underreporting and coverage exclusions. We expect that firefighters and law enforcement officers have higher levels of claim filing due to income, labor protections, knowledge, status, and proficiency with the medical and legal systems within which they work; other occupations may have lower filing rates [24] for a variety of reasons, including risking adverse consequences during the process [38]. However, it is unlikely that the stark differences in work-related injury and illness rates can be explained by reporting differences alone. Additionally, volunteer firefighters are not included in the WA WC system, and as such they are excluded from this analysis.

Administratively, workers’ compensation claims from self-insured employers and state fund employers may be different. In general, self-insured fire departments are larger, and the number and type of runs may be different than smaller departments. Compensable claim rates were elevated for firefighters, law enforcement officers and for ‘all other workers’ in self-insured employers when compared to state fund employers. There may also be differences in costs and days of time loss between state fund and self-insured claims, but because these data are not consistently reported to WA L&I, we cannot characterize them.

There were differences in injury type distribution between state fund and self-insured compensable claims. The majority of claims coded with a ‘nonclassifiable’ injury type were self-insured for all occupational groups. Whether there is differential injury type misclassification within the ‘nonclassifiable’ claims group is not known, but it is unlikely to diminish the overall significant excess of WMSDs in firefighters.

### 4.2. Strengths and Future Directions

This study used WA workers’ compensation data, which is a rich dataset to explore the differences between occupations, and includes extensive data on injured WA workers. The risk classification system allows us to calculate rates based on hours reported by employers for discrete occupational groups. While reporting of hours may also be incomplete because it is the mechanism by which premiums are paid, the process is regularly audited by WA L&I. We compare firefighters to law enforcement officers as another first-response occupation, as well as to the general worker population of WA (“all other” workers), giving a more complete picture of the health and safety of firefighters and their work-related injury and illness burden. Firefighters work in all states, although call volumes and types may vary by locality. We report decreasing claim rates over time and high WMSD risk, similar to OH and CA. Although this study only reports Washington’s experience, it provides an opportunity for other jurisdictions to evaluate if they have similar findings for this occupation.

While these descriptive analyses provide valuable insight for injury prevention and intervention activities and additional research, linking the WA employment records for firefighters to other data sources to better capture chronic illnesses and diseases with long latency, such as cancers [6], would be valuable. There is a need to better understand the injury, illness, and fatality rates in volunteer firefighters, and efforts should be made to characterize their experience. Additionally, potential differences in work-related injury and illness claims between municipal fire departments and fire districts (e.g., insurer type, funding, training), and between paid and volunteer firefighters, should be explored. Firefighters also have unique work schedules, which make comparison with other occupational groups difficult, and investigating the role of work organization (e.g., shift work) might yield insight into work-related injury and illness claim rates.

## 5. Conclusions

Firefighters face a variety of hazards in challenging situations. Using law enforcement officers and all other workers as comparison groups demonstrates the need for comprehensive risk management that takes into account these unique workplace hazards. While they have high rates of various specific work-related injuries and illnesses, a large portion of their claims are WMSDs. Work tasks placing firefighters at risk for WMSDs appear to be patient handling, use of tools and equipment, manual material handling, and physical training. Fire departments and community partners can use these results to evaluate their injury and illness data to identify avenues for prevention and intervention. Efforts to apply a risk management system directed to these work tasks could have a meaningful benefit for employers, firefighters and the communities they serve.

## Figures and Tables

**Table 1:** Annual average full-time equivalents (FTE) and compensable ^1^ workers’ compensation claims by insurance fund and occupation group, Washington State, 2006–2020.

	Firefighters	Law Enforcement Officers	‘All Other’ Workers
Average number of FTE per year	n (column %)	n (column %)	n (column %)
State fund	5248 (61.4)	3727 (36.6)	1,736,829 (72.4)
Self-insured	3301 (38.6)	6467 (63.4)	660,607 (27.6)
Total	8549 (100)	10,195 (100)	2,407,631 (100)
Average number of compensable ^1^ claims per year	n (column %)	n (column %)	n (column %)
State fund	274 (44.7)	145 (27.9)	25,099 (64.1)
Self-insured	338 (55.3)	375 (72.1)	14,030 (35.9)
Total	612 (100)	520 (100)	39,129 (100)
Total compensable^1^ claims, all years combined	n (column %)	n (column %)	n (column %)
State fund	4109 (44.7)	2174 (27.9)	376,486 (64.1)
Self-insured	5078 (55.3)	5627 (72.1)	210,453 (35.9)
Total	9187 (100)	7801 (100)	586,939 (100)
Compensable ^1^ claim rate	Rate per 10,000 FTE (95% CI)	Rate per 10,000 FTE (95% CI)	Rate per 10,000 FTE (95% CI)
State fund	521.9 (506.0, 537.9)	388.6 (372.3, 405.0)	144.5 (144.0, 145.0)
Self-insured	1025.6 (997.4, 1053.8)	579.9 (564.8, 595.1)	212.4 (211.5, 213.3)
Total	716.4 (701.7, 731.0)	510.0 (498.7, 521.3)	163.2 (162.8, 163.6)

FTE = Full-time equivalents (hours divided by 2000); CI = confidence interval. ^1^ Compensable claims are those that result in wage replacement/time loss, kept on salary, disability, or fatality.

**Table 2:** Demographic distribution of compensable ^2^ claim(s) by occupation group, Washington State, 2006–2020.

	Firefighters (n = 9187)	Law Enforcement Officers (n = 7801)	‘All Other’ Workers (n = 586,939)
Avg. number compensable ^2^ claims per claimant	1.8	1.5	1.3
Gender ^1^	(column %)	(column %)	(column %)
F	8.2	13.2	37.4
M	91.8	86.8	62.5
Age Group ^1^	(column %)	(column %)	(column %)
15–17	<1	–	<1
18–24	<1	<1	7.8
25–44	48.6	55.6	41.4
45–64	50.1	43.2	46.7
65–99	<1	<1	3.4
Marital Status ^1^	(column %)	(column %)	(column %)
Married	77.1	77.0	49.2
Single	21.9	22.0	50.0

^1^ Excludes ‘unknown’, ‘unknown or not specified’, ‘not defined in list’, or missing. ^2^ Compensable claims are those that result in wage replacement/time loss, kept on salary, disability, or fatality.

**Table 3:** Injury time and season for compensable ^1^ claims by occupation group, Washington State, 2006–2020.

	Firefighters	Law Enforcement Officers	‘All Other’ Workers
Total Number Compensable Claims ^1^	n = 9187	n = 7801	n = 586,939
Injury Time of Day ^2^	Column %	Column %	Column %
12:00–5:59 a.m.	7.3	9.3	4.9
6:00–11:59 a.m.	30.3	19.5	33.6
12:00–5:59 p.m.	28.2	28.6	31.6
6:00–11:59 p.m.	13.5	21.5	11.1
Missing/Unknown	20.7	21.2	18.8
Injury Season ^2^	Column %	Column %	Column %
Winter (December–February)	22.7	25.8	24.0
Spring (March–May)	24.6	25.0	24.5
Summer (June–August)	26.4	24.4	25.9
Fall (September–November)	26.3	24.8	25.6

^1^ Compensable claims are those that result in wage replacement/time loss, kept on salary, disability, or fatality. ^2^ Injury time and date refer to when the exposure happened that led to the injury or illness for acute conditions. For chronic conditions, the injury time and date refer to the last exposure that led to the injury or illness.

**Table 4:** Compensable ^1^ claim events by occupational group, Washington State, 2006–2020.

	Firefighters		Law Enforcement Officers		‘All Other’ Workers	
Event Type ^2^ (OIICS v1.01 Codes)	Rate per 10,000 FTE (95% CI)	Column % (n)	Rate per 10,000 FTE (95% CI)	Column % (n)	Rate per 10,000 FTE (95% CI)	Column % (n)
WMSD ^2^	277.0 (267.9, 286.1)	38.7 (3552)	76.2 (71.9, 80.6)	14.9 (1166)	57.6 (57.4, 57.9)	35.3 (207,192)
Struck by/against; caught in/under/between (010–049)	52.4 (48.4, 56.4)	7.3 (672)	35.2 (32.3, 38.2)	6.9 (539)	24.5 (24.4, 24.7)	15.0 (88.235)
Fall to lower level (11 *)	21.6 (19.1, 24.1)	3.0 (277)	12.5 (10.7, 14.3)	2.4 (191)	10.3 (10.2, 10.4)	6.3 (37,097)
Fall to same level (13 *)	33.3 (30.1, 36.5)	4.6 (427)	40.2 (37.0, 43.3)	7.9 (614)	17.5 (17.4, 17.7)	10.7 (63,011)
Slip, trip, loss of balance without fall (215)	18.6 (16.3, 21.0)	2.6 (239)	12.6 (10.8, 14.4)	2.5 (193)	4.7 (4.6, 4.8)	2.9 (16,833)
Bodily reaction, N.E.C. (219)	105.2 (99.6, 110.8)	14.7 (1349)	70.2 (66.0, 74.4)	13.8 (1074)	8.6 (8.5, 8.7)	5.3 (30,998)
Running or walking without other incident (213, 217)	10.4 (8.7, 12.2)	1.5 (134)	14.6 (12.7, 16.6)	2.9 (224)	0.9 (0.9, 1.0)	0.6 (3381)
Contact with electric current, temperature extremes, or exposure to air pressure changes (31 *,32 *,33 *)	3.6 (2.6, 4.6)	0.5 (46)	0.9 (0.4, 1.3)	0.2 (13)	1.3 (1.3, 1.3)	0.8 (4649)
Exposure to caustic, noxious, or allergenic substances (34 *)	43.6 (40.0, 47.2)	6.1 (559)	10.1 (8.5, 11.7)	2.0 (155)	2.7 (2.6, 2.7)	1.6 (9646)
Exposure to noise (35 *)	13.5 (11.5, 15.5)	1.9 (173)	14.6 (12.7, 16.5)	2.9 (223)	2.3 (2.3, 2.4)	1.4 (8401)
Transportation accidents (4 *)	9.7 (8.0, 11.4)	1.3 (124)	49.8 (46.2, 53.3)	9.8 (761)	5.2 (5.1, 5.3)	3.2 (18,725)
Fires and explosions (5 *)	3.6 (2.6, 4.6)	0.5 (46)	–	–	0.1 (0.1, 0.1)	0.1 (420)
Assaults and violent acts (6 *)	3.0 (2.1, 4.0)	0.4 (39)	96.2 (91.3, 101.1)	18.9 (1471)	4.1 (4.0, 4.1)	2.5 (14,590)
All other	88.7 (83.6, 93.9)	12.4 (1138)	49.1 (45.6, 52.6)	9.6 (751)	16.6 (16.5, 16.7)	10.2 (59,713)
Non-classifiable	32.1 (29.0, 35.2)	4.5 (412)	27.7 (25.1, 30.4)	5.4 (424)	6.7 (6.6, 6.8)	4.1 (24,048)

OIICS = Occupational Injury and Illness Classification System; * indicates all codes in section are included. ^1^ Compensable claims are those that result in wage replacement/time loss, kept on salary, disability, or fatality. ^2^ All categories here are mutually exclusive. Events leading to compensable claims described here using OIICS v1.01 event groups. The WMSD category is an exception and is based on OIICS event and nature codes. See Ref [5] for more detail. Rates suppressed when based on <5 claims.

**Table 5:** Compensable ^1^ COVID-19 exposure ^2^ and Post-Traumatic Stress Disorder (PTSD) ^3^/Post-traumatic Anxiety (PTA) ^4^ claims by occupational group, Washington State, 2018–2020.

	Firefighters	Law Enforcement Officers	‘All Other’ Workers
Special conditions of interest	Rate per 10,000 FTE (95% CI)	Rate per 10,000 FTE (95% CI)	Rate per 10,000 FTE (95% CI)
COVID-19 exposure ^2^	321.7 (277.9, 365.5)	119.5 (85.3, 153.7)	26.5 (25.8, 27.3)
PTSD ^3^	32.9 (24.6, 64.9)	40.0 (28.6, 51.4)	–
PTA ^4^	10.3 (5.6, 14.9)	7.7 (2.7, 12.7)	–

^1^ Compensable claims are those that result in wage replacement/time loss, kept on salary, disability, or fatality. ^2^ Claims for occupational exposure to COVID-19 virus were identified using a OIICS codes, ICD-9-CM diagnosis codes, and keywords. COVID-19 claims are only described for 2020. ^3^ Post-traumatic stress disorder (PTSD) claims were identified using an administrative flag in the WA workers’ compensation system to identify claims adjudicated under the presumption law starting in 2018 (2018–2020 data). Workers’ compensation coverage for primary PTSD is only available to firefighters and law enforcement officers. ^4^ Post-traumatic anxiety (PTA) claims were identified by OIICS v1.01 codes for nature of injury or illness 5211 and 5212: “post-traumatic anxiety-acute” and “post-traumatic anxiety-chronic” (2018–2020 data). Workers’ compensation coverage for primary PTA is only available to firefighters and law enforcement officers.

**Table 6:** Claim costs and time loss associated with compensable ^1^ claims by occupation group, state fund only ^2^, Washington State, 2006–2020.

	Firefighters	Law Enforcement Officers	‘All Other’ Workers
Total claims, state fund ^2^	n = 4109	n = 2174	n = 376,486
% of claims due to WMSDs ^3^, state fund ^2^	1642 (40%)	331 (15.2)	125,827 (33.4)
Total ^4^ claim costs (US$)			
Percentile: 25	2882	3735	2556
Percentile: 50 (median)	8286	11,506	9307
Percentile: 75	25,502	31,471	33,330
Average (mean)	46,318	46,789	46,645
Total ^4^ WMSD claim costs (US$)			
Percentile: 25	2889	3736	2985
Percentile: 50 (median)	7651	11,544	11,293
Percentile: 75	25,526	34,106	40,122
Average (mean)	31,169	42,266	50,294
Medical costs (US$)			
Percentile: 25	1298	1989	1850
Percentile: 50 (median)	3927	6115	6022
Percentile: 75	11,125	15,592	17,266
Average (mean)	13,836	14,673	17,670
WMSD Medical costs (US$)			
Percentile: 25	1264	2042	2130
Percentile: 50 (median)	3442	6292	7037
Percentile: 75	10,778	15,915	19,265
Average (mean)	9417	13,657	17,460
Time loss days ^5^			
Percentile: 25	10	9	13
Percentile: 50 (median)	28	26	49
Percentile: 75	76	78	220
Average (mean)	110	133	287
WMSD time loss days ^5^			
Percentile: 25	10	10	15
Percentile: 50 (median)	29	32	61
Percentile: 75	79	82	289
Average (mean)	105	153	321

^1^ Compensable claims are those that result in wage replacement/time loss, kept on salary, disability, or fatality. ^2^ Only state fund claims are included in cost and time loss days analyses, self-insured data for these metrics are incomplete. ^3^ Work-related musculoskeletal disorders (WMSDs) definition based on Occupational Injury and Illness Classification System (OIICS) nature and event codes v1.01. See Ref [5]. ^4^ Total claim costs include medical costs, indemnity or wage replacement payments, and all other costs incurred by the claim. ^5^ Only compensable claims with time loss are included in this calculation. Some compensable claims, such as those involving a fatality or kept on salary, do not have time loss associated with them.

**Table 7:** Common circumstances of compensable ^1^ WMSD ^2^ claims among firefighters, Washington State, 2006–2020.

OIICS v1.01 Source of Injury or Illness	n (col %)	Common Tasks/Situations ^3^
Healthcare patient or resident of healthcare facility	1038 (29.2)	Lifting patients—from floors/transfer equipment; in hospitals, residences, hazardous sites; moving patients—rolling, dragging, carrying; navigating stairs, slopes, clutter/debris; using gurneys, stretchers, stokes baskets; vehicles (cars, boats); morbidly obese patients
Bodily motion or position of injured, ill worker	501 (14.1)	Bending down; climbing—ladders, into vehicles/apparatus; crawling; using/hauling hoses; carrying patients/gear; physical training (exercise)
Hoses	371 (10.4)	Hose handling—lifting, pulling, charging, carrying; in training and in hazardous locations
No description at this level	361 (10.2)	Lifting, carrying, bending; forcible entries; confined spaces; operating or moving equipment/gear; physical training (exercise)
Healthcare and orthopedic equipment, N.E.C.	196 (5.5)	Transporting patients—gurneys, stretchers, backboards; in/out of: ambulances, other vehicles, steps, airlift, trail
Gymnasium and exercise equipment	116 (3.3)	Physical training, more detailed descriptions—weightlifting, specific exercise machines
Tools, instruments and equipment, N.E.C.	75 (2.1)	Hooks, poles, training mannequins, drills, haligons, SCBA gear, axes, ladders, air packs, other heavy equipment
Ladders–moveable	71 (2.0)	Lifting, carrying, extending
Ladders, unspecified	51 (1.4)	Lifting, raising, pulling
Truck	41 (1.2)	Driving at speed (jostling, jarring, speed bumps, etc.); opening/closing doors
Person—other than injured or ill worker, N.E.C.	40 (1.1)	Lifting, dragging, and/or carrying people
Cutting hand tools—powered	38 (1.1)	Various saws and other extraction tools
Boxes, crates, cartons	33 (<1)	Lifting, carrying, and moving boxes—storage, supplies, medical boxes, tool boxes
Bags, sacks, totes	32 (<1)	Firefighting gear; medical equipment; physical training (exercise)
Recreation and athletic equipment, N.E.C.	32 (<1)	Physical training, more generic descriptions—weights, workout
All other sources	556 (15.7)	
Total	3552 (100)	

OIICS = Occupational Injury and Illness Classification System; N.E.C. = not elsewhere classifiable. ^1^ Compensable claims are those that result in wage replacement/time loss, kept on salary, disability, or fatality. ^2^ Work-related musculoskeletal disorders (WMSDs) definition-based OIICS nature and event codes v1.01. See Ref [5]. ^3^ Excerpts from the ‘injury description’ field of the Report of Industrial Injury or Occupational Disease form.

## Data Availability

The data presented in this study are available on a limited basis (aggregate, de-identified) upon request from the corresponding author. The data are not publicly available due to legal, privacy, and ethical restrictions.

## References

[1] Poplin G.S., Griffin S., Pollack Porter K., Mallett J., Hu C., Day-Nash V., Burgess J.L. (2018). Efficacy of a proactive health and safety risk management system in the fire service. Inj. Epidemiol..

[2] Phelps S.M., Drew-Nord D.C., Neitzel R.L., Wallhagen M.I., Bates M.N., Hong O.S. (2018). Characteristics and Predictors of Occupational Injury Among Career Firefighters. Workplace Health Saf..

[3] International Agency for Research on Cancer (IARC) (2023). Occupational Exposure as a Firefigher.

[4] Le A.B., McNulty L.A., Dyal M.-A., DeJoy D.M., Smith T.D. (2020). Firefighter Overexertion: A Continuing Problem Found in an Analysis of Non-Fatal Injury Among Career Firefighters. Int. J. Environ. Res. Public Health.

[5] Marcum J. (2022). Workers' Compensation Claims Due to Musculoskeletal Disorders, Washington State, 2006–2019.

[6] LaSee C., Bonauto D., Marcum J. (2022). Workers' compensation claims for conditions presumed to be occupational diseases among firefighters in Washington State, 2000–2017. J. Occup. Enivron. Med..

[7] Washington State Council of Firefighters (2019). Healthy In, Healthy Out: Best Practices for Reducing Fire Fighter Risk of Exposures to Carcinogens.

[8] Pollack K.M., Poplin G.S., Griffin S., Peate W., Nash V., Nied E., Gulotta J., Burgess J.L. (2017). Implementing risk management to reduce injuries in the U.S. Fire Service. J. Saf. Res..

[9] State of Washington (2019). Concerning Firefighter Safety.

[10] National Academy of Social Insurance (2021). Workers' Compensation: Benefits, Coverage, and Costs (2019 Data).

[11] (1986). General Provisions.

[12] (2022). Employments excluded.

[13] Washington State Department of Labor & Industries (2021). L&I Facts and Figures.

[14] State of Washington (2021). Washington Administrative Code WAC 296-17—General Reporting Rules, Audit and Recordkeeping, Rates and Rating System for Washington Workers' Compensation Insurance.

[15] U.S. Fire Administration National Fire Department Registry. https://apps.usfa.fema.gov/registry/.

[16] U.S. Department of Labor—Bureau of Labor Statistics (2007). Occupational Injury and Illness Classification Manual.

[17] Todorov D., Reeb-Whitaker C. (2021). COVID-19 Surveillance in Washington Workers' Compensation Data: March 2020 to June 2021.

[18] Sritharan J., Kirkham T.L., MacLeod J., Marjerrison N., Lau A., Dakouo M., Logar-Henderson C., Norzin T., DeBono N.L., Demers P.A. (2022). Cancer risk among firefighters and police in the Ontario workforce. Occup. Environ. Med..

[19] Quinn T.D., Marsh S.M., Oldham K., Wurzelbacher S.J., Naber S.J. Workers' compensation injury claims among firefighters in Ohio, 2001–2017. J. Saf. Res..

[20] Ruggles S., Flood S., Goeken R., Schouweiler M., Sobek M. (2022). IPUMS USA: Version 12.0 [Dataset]—ACS Estimates for Washington State.

[21] LEOFF Plan 2 Retirement Board. Demographics & FAQs. https://leoff.wa.gov/member-resources/demographics-faqs.

[22] Fahy R., Evarts B., Stein G.P., National Fire Protection Association (NFPA) (2022). US Fire Department Profile 2020.

[23] CDC-NIOSH Employed Labor Force (ELF) Estimates—Washington State, 2015–2019. https://wwwn.cdc.gov/wisards/cps/default.aspx.

[24] Fan Z.J., Bonauto D.K., Foley M.P., Silverstein B.A. (2006). Underreporting of Work-Related Injury or Illness to Workers' Compensation: Individual and Industry Factors. J. Occup. Enivron. Med..

[25] Lipscomb H.J., Loomis D., McDonald M.A., Arguc R.A., Wing S. (2006). A Conceptual Model of Work and Health Disparities in the United States. Int. J. Health Serv..

[26] Smith P.M., Kosny A.A., Mustard C.A. (2009). Differences in Access to Wage Replacement Benefits for Absences Due to Work-Related Injury or Illness in Canada. Am. J. Ind. Med..

[27] Kyung M., Lee S.-J., Dancu C., Hong O.S. (2023). Underreporting of workers' injuries or illnesses and contributing factors: A systematic review. BMC Public Health.

[28] Scherzer T., Rugulies R., Krause N. (2005). Work-Related Pain and Injury and Barriers to Workers' Compensation Among Las Vegas Hotel Room Cleaners. Am. J. Public Health.

[29] Smith J.D.R. (2012). Immigrant Workers and Worker's Compensation: The Need for Reform. Am. J. Ind. Med..

[30] Dworsky M., Seabury S.A., Broten N. (2021). The Frequency and Economic Impact of Musculoskeletal Disorders for California Firefighters. Rand Health Q..

[31] Turner J.A., Franklin G., Fulton-Kehoe D., Sheppard L., Wickizer T.M., Wu R., Gluck J.V., Egan K. (2006). Worker recovery expectations and fear-avoidance predict work disability in a population-based workers' compensation back pain sample. Spine.

[32] Besen E., Young A.E., Shaw W.S. (2015). Returning to work following low back pain: Towards a model of individual psychosocial factors. J. Occup. Rehabil..

[33] Quartana P.J., Campbell C.M., Edwards R.R. (2009). Pain catastrophizing: A critical review. Expert Rev. Neurother..

[34] Scheelar J.F. (2002). A return to worker role after injury: Firefighters seriously injured on the job and the decision to return to high-risk work. Work.

[35] Hanson B., Steele Cooper S., Tegarden T., Tipton L., Freeman A.M., Davis K.G., Gillespie G.L., Huston T. (2021). The impact of emergency responder musculoskeletal injuries in the State of Ohio. Work.

[36] The Office of the Governor, Washington State (2020). Inslee Announces Workers' Compensation Coverage to Include Quarantined Health Workers/First Responders.

[37] (2019). State of Washington. Revised Code of Washington, RCW 51.32.185: Occupational diseases—Presumption of occupational disease for firefighters and fire investigators—Limitations—Exception—Rules—Advisory committee on occupational disease presumptions. Legislature, W.S., Ed..

[38] Azaroff L.S., Levenstein C., Wegman D.H. (2002). Occupational Injury and Illness Surveillance: Conceptual Filters Explain Underreporting. Am. J. Public Health.

